# *In situ* Preparation of Chitosan/ZIF-8 Composite Beads for Highly Efficient Removal of U(VI)

**DOI:** 10.3389/fchem.2019.00607

**Published:** 2019-09-06

**Authors:** Lijuan Liu, Weiting Yang, Dongxu Gu, Xiaojun Zhao, Qinhe Pan

**Affiliations:** ^1^Key Laboratory of Advanced Materials of Tropical Island Resources, Ministry of Education, School of Science, Hainan University, Haikou, China; ^2^Hainan Policy and Industrial Research Institute of Low-Carbon Economy, Hainan University, Haikou, China

**Keywords:** chitosan, ZIF-8, composite, uranium, adsorption

## Abstract

With the rapid growth of nuclear power generation and fuel processing, the treatment of nuclear industry wastewater has become a major problem, and if not handled properly, it will pose a potential threat to the ecological environment and human health. Herein, a chitosan (CS)/ZIF-8 composite monolithic beads with ZIF-8 loading up to 60 wt% for U(VI) removal was prepared, which can be easily removed after use. It possesses a very high adsorption capacity of 629 mg•g^−1^ at pH = 3 for U(VI) and a well recyclability is demonstrated for at least four adsorption/desorption cycles. X-ray photoelectron spectroscopy (XPS) was carried out to study the adsorption mechanism between uranium and adsorbent, and the chelation of U(VI) ions with imidazole, hydroxyl, and amino groups was revealed. This work shows that CS/ZIF-8 composite can be used as an effective adsorbent for uranium extraction from aqueous solution, and has a potential application value in wastewater treatment.

## Introduction

Along with the continuous development of industrial modernization, the demand for nuclear energy is rapidly increasing owing to its high energy density and greenhouse gas-free emission. Uranium is a typical core resource in nuclear reaction. It is radioactive and highly toxic, and has a high carcinogenicity (Li et al., 2016). Once discharged into the environment, it will lead to serious pollution to the water body (Fu et al., 2017). While getting inside the human body, it will cause irreversible damage to the internal organs (Zhang M. C. et al., 2018). Therefore, from the perspective of environmental protection and human health, it is particularly important to recover uranium efficiently from aqueous solution. At present, many techniques for uranium recovery from aqueous solution have been developed, such as photocatalytic method (Li Z. J. et al., 2017; Deng et al., 2019), chemical extraction (Sadeghi et al., 2012; Carboni et al., 2013; Wang et al., 2015), chemical flocculation method (Newsome et al., 2015), and adsorption method (Huang et al., 2018). Among these, adsorption method is one of the most extensive technologies because of low cost, simple operation, high efficiency, and good removal effect (Li et al., 2018a; Wang L. et al., 2018). The adsorbents adopted in the uranium adsorption technique include oxides (Yu et al., 2013), sulfides (Manos and Kanatzidis, 2012), hydroxides (Li R. et al., 2017), poly (amid oxime) and its derivatives (Wang D. et al., 2018), carbon nanotubes (Chen et al., 2018), phosphates (Zheng et al., 2015; Cai et al., 2017), porous silica (Huynh et al., 2017), and porous carbon (Starvin and Rao, 2004) etc. However, most of the adsorbents have some disadvantages, like low adsorption capacity, poor stability, or inability to circulate etc. So developing highly efficient uranium adsorbent materials is still in needed.

Recently, metal-organic frameworks (MOFs), as a class of novel porous material with high surface area (He et al., 2016; Zhao et al., 2018; Li et al., 2019a), adjustable pore size (Zou et al., 2009; Luo et al., 2016; Cheng et al., 2018), and high porosity (Luo et al., 2018; Sun et al., 2018; Li et al., 2019b), have attracted extensive attentions in various fields (Fang et al., 2007; Banerjee et al., 2008). Regarding to uranium separation and recovery, some MOFs and MOF-based composites have been developed (Liu et al., 2018; Yang et al., 2019). For example, Wang et al. demonstrated, for the first time, that the multilayered V_2_CTx MXene could be used as a potential and efficient adsorbent for uranium capture from aqueous solution (Wang et al., 2016). Yang et al. reported using a rare earth-based MOF material, MOF-76, for luminescent sensing and adsorption of uranium (Yang et al., 2013). The adsorption was evaluated up to 298 mg•g^−1^ at a relatively low pH of 3.0 ± 0.1. In general, such crystalline materials always lack flexibility and process ability, which limits their application to actual uranium adsorption (Kitao et al., 2017). Combining MOFs and polymers to prepare composite monoliths will provide beneficial and significant improvement while maintaining high adsorption capacity and providing convenient recycling. Wang et al. processed MOFs into nanofiber filters, which can selectively adsorb toxic SO_2_ gas when exposed in a SO_2_/N_2_ mixture stream (Zhang et al., 2016). Li et al. fabricated a high-quality ZIF-8/PSS membrane, which showed excellent performance in the nanofiltration and separation of dyes from water (Zhang et al., 2014). For uranium separation, Wang et al. prepared the only example of a ZIF-8 based polyacrylonitrile (PAN) fibrous filter, which removed uranyl ions efficiently (Wang C. H. et al., 2018). Thus, more detailed investigations for this target are desirable.

Natural polymers are widely concerned by various industries due to their biocompatibility, biodegradability, non-toxicity, adsorption performance, low cost, etc. (Lee et al., 2011). Chitosan (CS) is an important renewable natural biomass. There are lots of free amino and hydroxyl groups in its structure, which is advantageous to various chemical modifications and hybridization. Owing to such features, chitosan and its composites have been widely used for anti-bacterial coating, drug delivery, wound dressing, and cartilage regeneration (Mohammadzadeh Pakdel and Peighambardoust, 2018). For example, Wang et al. investigated the U(VI) adsorption behavior on cross-linked chitosan (Wang et al., 2009). Zhang et al. developed an impregnation-gelation-hydrothermal technique to prepare hybrid microspheres and hollow fibers consisting of zeolites and chitosan, which could serve as effective absorbents to remove Cu(II) (Zhang Y. Y. et al., 2018). To the best of our knowledge, there is no report on chitosan composites with MOF for uranium adsorption or separation.

**Scheme 1:**
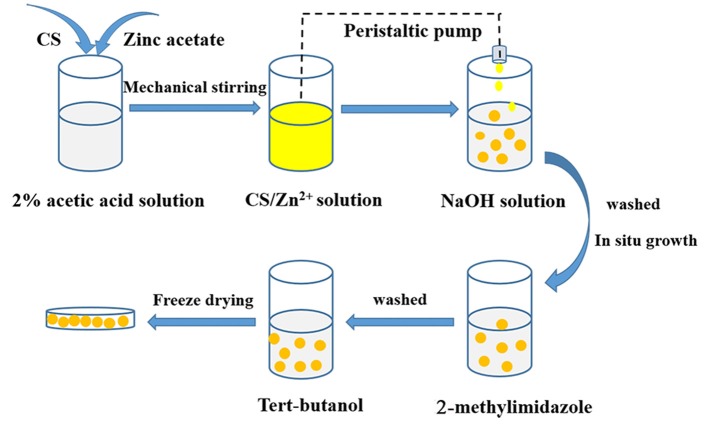
Schematic depicting the *in situ* preparation of CS/ZIF-8 composite beads.

Based on the above considerations, in this paper, the *in situ* synthesis of a CS/ZIF-8 composite was developed (Scheme 1). Chitosan/zinc ions beads were prepared using a peristaltic pump firstly. When the zinc ions-containing chitosan beads were in contact with the 2-methylimidazole solution, ZIF-8 nanocrystals grew to form the CS/ZIF-8 composite beads, which could recover U(VI) from aqueous solution. The effects of pH, concentration, and adsorption time on its adsorption performances were studied as well as the probable mechanism.

## Experiment

### Materials

Chitosan (CS) was purchased from Shanghai Aladdin Biochemical Technology Co., Ltd. (Shanghai, China); UO_2_(NO_3_)_2_·6H_2_O was purchased from Hubei Chu Sheng Wei Chemistry Co., Ltd.; Deionized water was used in all experiments.

### Preparation of CS/ZIF-8 Composite Beads

**Figure 1:**
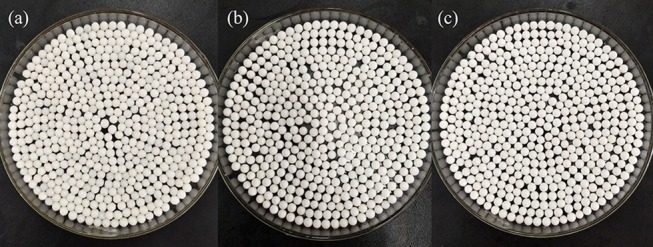
Optical photos of CS/ZIF-8 composite beads with different content of ZIF-8 **(A)** CS/ZIF-8–36%; **(B)** CS/ZIF-8–51%; **(C)** CS/ZIF-8–60%.

The preparation process of CS/ZIF-8 composite beads is shown in Scheme 1. 3.0 g chitosan and 1.487 g zinc acetate were dissolved into 0.1 L acetic acid solution (2.0%, v/v) with stirring at 550 rpm for 4 h to form a homogeneous solution. Then, the solution was dripped into 1 M NaOH with a peristaltic pump. After 20 min, the CS/Zn^2+^ microspheres were taken out and washed for 3 times with deionized water to remove away excess NaOH, and then they were soaked in an aqueous solution containing 2.315 g (0.15 mol) 2-methylimidazole. At this time, Zn^2+^ would react with 2-methylimidazole to form ZIF-8 in the microsphere matrix. Next, the obtained CS/ZIF-8 hydrogel composite beads were washed with deionized water for 3 times, soaked in tert-butanol, changed fresh solution every 20 min, subsequently freeze-dried for 12 h to get CS/ZIF-8 composite beads (Figure 1). The average dimension of the prepared composite beads is about 2.5 mm in diameter. The ZIF-8 content in the CS/ZIF-8 composites can be adjusted by changing the initial Zn(CH_3_COO)_2_ amounts.

### Characterization

Fourier transform infrared (FT-IR) spectroscopy was conducted by Bruker TENSOR27. The morphology was investigated by a scanning electron microscope (Hitachi S-4800). Powder X-ray diffraction (PXRD) data were obtained by Miniflex-600, with Cu Kα radiation at 40 kV and 15 mA. The thermos gravimetric (TG) curves within 30–800°C were collected on a TA Q600 instrument under air flow. The concentration of U(VI) was determined by the arsenazo III spectrophotometric method, which was measured on a UV spectrophotometer (UV-1801, Beijing Beifen Rayleigh Analytical Instruments (Group) Co., Ltd.). The uranium and the interfering elements concentration were measured by ICP-OES (X Series, Thermo Fisher, USA). The nitrogen adsorption/desorption experiment was conducted at 77 K (ASAP2020M+c, Micromeritics Instrument Corporation, USA). The X-ray photoelectron spectroscopy (XPS) spectra were obtained by using ESCALAB 250Xi (Thermo Fisher, USA) with Al Kα radiation at 1,253.6 eV.

### Batch Adsorption Experiments

In a general procedure, 0.02 g of UO_2_ (NO_3_)_2_·6H_2_O was dissolved in 0.1 L deionized water to obtain a stock solution. The test solutions were prepared by diluting the U(VI) stock solution. The pH was adjusted by 0.1 M NaOH or HCl solution. CS/ZIF-8 composite beads (0.002 g) were added into 0.01 L solution of U(VI). The mixture was shaken at room temperature for desired reaction time. The concentration of U(VI) was determined by the arsenazo III spectrophotometry. The control experiments were conducted under similar conditions: only ZIF-8 powder or CS replaced the CS/ZIF-8 composites. The U(VI) adsorption capacity (*q*_e_) of the samples was calculated according to Equation 1 (Song et al., 2018):

(1) $$\begin{array}{r} q_{e}=\frac{\left(C_{0}-C_{e}\right)V}{m} \end{array}$$

where *C*_0_ refers to the U(VI) initial concentration (mg•L^−1^), *C*_e_ is the equilibrium concentration (mg•L^−1^), *V* (L) refers to the solution volume, and *m* (g) is the weight of the adsorbent.

After adsorption, the uranium-loaded CS/ZIF-8 adsorbents were used directly for the elution test. The eluate was collected after shaking for 20 min on a shaker using 0.02 L of a solution containing 0.1 M NaHCO_3_ as an eluent, then the uranium concentration in the eluate was analyzed. Then the CS/ZIF-8 adsorbents were washed with circulating deionized water once before being used next for uranium adsorption-desorption cycles, which followed the same procedure as described above.

## Results and Discussion

### Characterization of the CS/ZIF-8 Composite Beads

**Figure 2:**
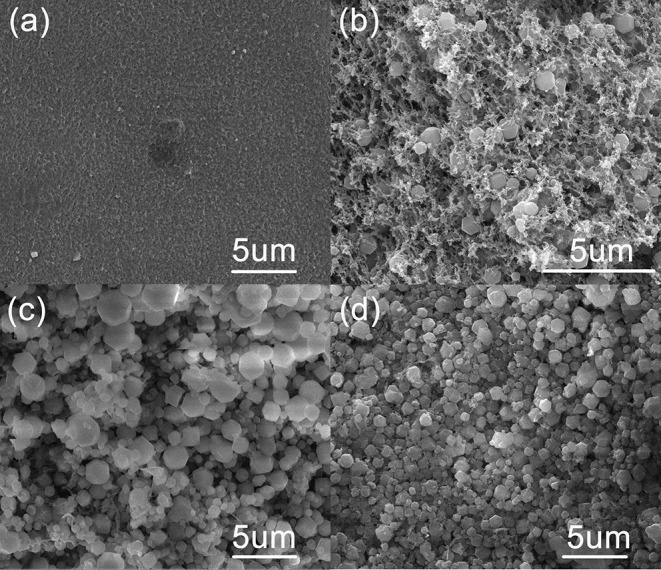
SEM images of CS/ZIF-8 composite beads with different content of ZIF-8. **(A)** pure CS; **(B)** CS/ZIF-8–36%; **(C)** CS/ZIF-8–51%; **(D)** CS/ZIF-8–60%.

The prepared composite beads are uniform with average size of 2.5 mm in diameter, which are very stable and easy to store. Scanning electron microscopy (SEM) images show the surface features and interfacial interactions of pure CS and CS/ZIF-8 composites. As shown in Figure 2A, the surface of pure CS material exhibits a smooth and evenly porous pattern. After composition with ZIF-8, the surface becomes rough due to the attachment of many ZIF-8 nanoparticles (Figure 2B), whose dodecahedral morphologies are clearly visible. As the content of Zn^2+^ increases in the initial reaction mixture, more ZIF-8 nanoparticles grow on the surface and internal of chitosan, and the size is getting smaller (Figures 2C,D).

**Figure 3:**
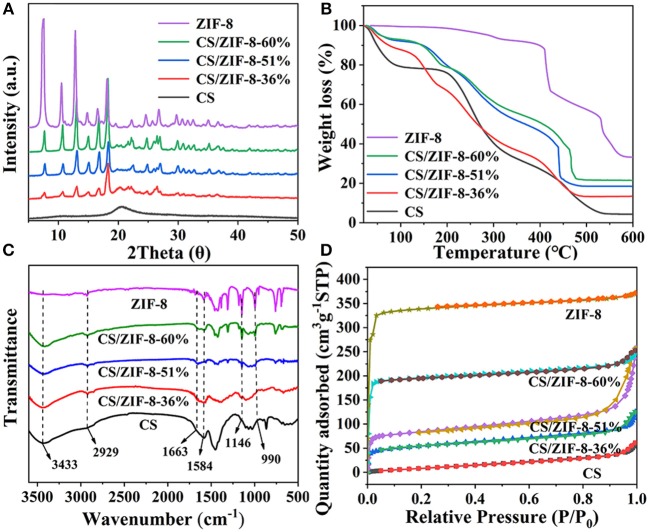
**(A)** PXRD patterns, **(B)** TG curves, **(C)** FT-IR spectra, **(D)** Nitrogen adsorption/desorption isotherms of CS, ZIF-8, CS/ZIF-8–36%, CS/ZIF-8–51%, CS/ZIF-8–60%.

The PXRD patterns further confirm the successful growth of ZIF-8 within the CS beads (Figure 3A). Due to the small content of ZIF-8 in the early stage, the peak of ZIF-8 is relatively weak. With the content of ZIF-8 increasing, the peak intensity gradually enhanced. In order to determine the stability of the CS/ZIF-8 composites under acidic or alkaline conditions, the composite beads were soaked in the solution with different pH (3 to 13). Three days later, the PXRD patterns of the composite beads were measured and no change was found, revealing the good stability at the pH condition (Figure S1).

In order to know the content of ZIF-8 in the composite beads, ICP analysis was performed, giving the ZIF-8 content of 36, 51, and 60 wt% corresponding to zinc acetate initial amount of 1.487, 2.975, and 4.462 g, respectively. For convenience, the samples with these different ZIF-8 loading are denoted as CS/ZIF-8–36%, CS/ZIF-8–51%, CS/ZIF-8–60%. As shown in Figure 3B, the thermal degradation of chitosan occurs in three steps: before 100°C, there is a small weight loss process about 10%, which is caused by the bound water and crystal water contained in the material. This process is an endothermic reaction. At 220–300°C, chitosan is strongly degraded, with a weight loss of about 50%. At 300–600°C, the degradation is slow, the weight loss is about 40%. Both steps of thermal degradation are exothermic reactions and the thermal degradation ends at 600°C. ZIF-8 has a residual of 33% at 600°C, which is consistent with the theoretical value (35%). With the increase of ZIF-8 content, the thermal decomposition temperature gradually rises, indicating the existence of some interaction between CS and ZIF-8 (Figure S2). Together with the TG analyses of CS/ZIF-8 composites, we can also verify the loading of ZIF-8 in CS/ZIF-8 composite beads, that is in agreement with ICP results.

FT-IR spectroscopy is shown in Figure 3C. For CS, -OH groups vibrate at a wide band of 3,433 cm^−1^, overlapping with -NH stretching vibration. The characteristic peak at 1,660 cm^−1^ corresponds to the vibrations of the –NH_2_ group. Specifically, the absorption peak at 2,929 cm^−1^ is ascribed to the C-H bond stretching vibration from the methyl imidazole ring of ZIF-8. The absorption peak at 1,584 cm^−1^ belongs to the C = N vibrations, while peaks at 1,146 and 990 cm^−1^ are from C-N stretching vibration. In addition, from these spectra, we can see that with the increasing of ZIF-8 content, the characteristic absorption peaks of ZIF-8 in composites are enhanced.

The specific surface areas of the CS/ZIF-8 composites were determined by nitrogen adsorption. The N_2_ absorption/desorption isotherms show that all materials exhibit a typical I-type isotherm with micropore character (Figure 3D). With the increase of ZIF-8 content, the specific surface area also increases sequentially, which is 184.93, 279.24, and 628.80 m^2^•g^−1^, respectively, for three different ZIF-8 loading composites. The specific surface area of pure ZIF-8 is 1080.91 m^2^•g^−1^, while only 40.07 m^2^•g^−1^ for pure CS beads. This provides possibility of the CS/ZIF-8 composite beads for efficient adsorption of U(VI).

### Evaluation of U(VI) Adsorption Performance

#### Effect of Initial pH

**Figure 4:**
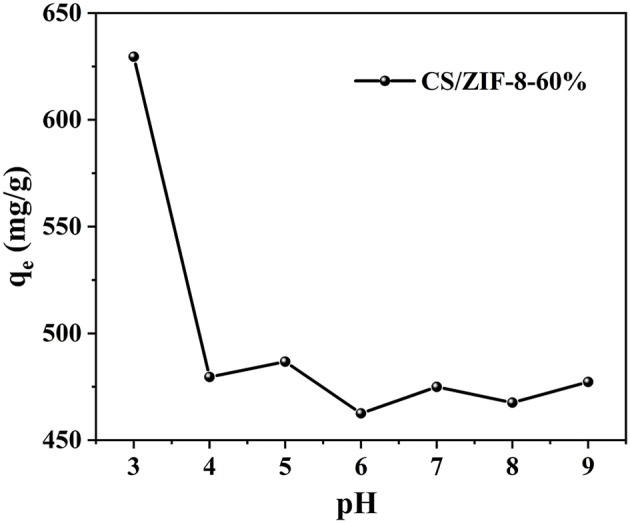
Effect of pH on U(VI) adsorption of CS/ZIF-8–60% composite beads (*C*_0_ = 100 mg•L^−1^, t = 24 h, *m*_ads_/*V*_sol_ = 0.2 g•L^−1^).

**Figure 5:**
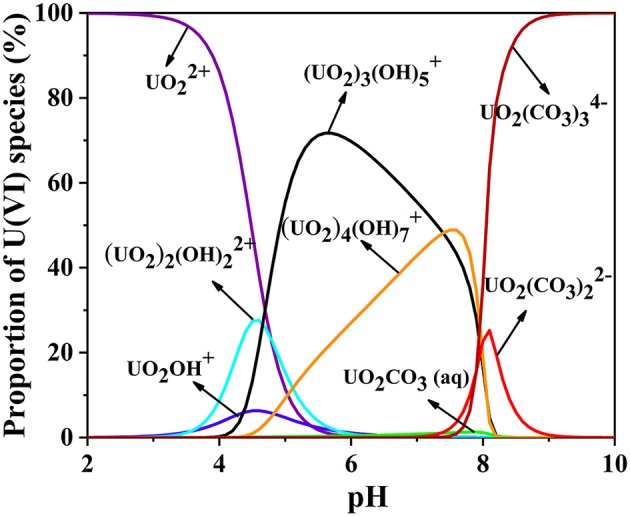
The pH-dependence of various U(VI) species in aqueous solution C_*U*(*VI*)_ = 100 mg L^−1^ (0.038% atm in the presence of CO_2_).

pH is an important parameter in uranium batch adsorption experiments (Zhang et al., 2017), due to its dramatic influence on the charge and active site of the sorbent and the speciation of U(VI) in solution (Min et al., 2017). Chitosan dissolves under acidic condition of pH = 2. Therefore, a series of experiments have been performed on the CS/ZIF-8 composite beads under pH values ranging from 3 to 9. As shown in Figure 4, the maximum adsorption capacity of U(VI) is obtained as 629 mg•g^−1^ at pH = 3.0, and then gradually decreases as the pH increases. This is a similar trend to the work reported previously where Fe_3_O_4_@ZIF-8 (Min et al., 2017) and ZIF-8/PAN (Wang C. H. et al., 2018) were investigated for the adsorption of uranium. As shown in Figure 5, at pH of 3, U(VI) mainly exits in the form of ${\text{UO}}_{2}^{2+}$ cation, as the pH increases, it will be hydrolyzed to oligomeric or colloidal species, such as (UO_2_)_3_${\left(\text{OH}\right)}_{5}^{+}$, (UO_2_)_4_${\left(\text{OH}\right)}_{7}^{+}$, (UO_2_)_2_${\left(\text{OH}\right)}_{2}^{2+}$, and UO_2_(OH)^+^ etc. (Chen et al., 2018). Due to the increased dimensions of these species, a decrease of adsorption efficiency is resulted with pH increasing (Wang C. H. et al., 2018). In addition, the decreased uptake trends at pH > 6.5 may also arise from the electronic repulsion between the negative charged U(VI) species including UO_2_${\left({\text{CO}}_{3}\right)}_{2}^{2-}$ and UO_2_${\left({\text{CO}}_{3}\right)}_{3}^{4-}$ and the adsorbent surfaces (Cai et al., 2017). So pH of 3 is the optimal adsorption value, and following adsorption investigations were performed at this condition.

#### Adsorption Kinetics of the CS/ZIF-8 Composite Beads

**Figure 6:**
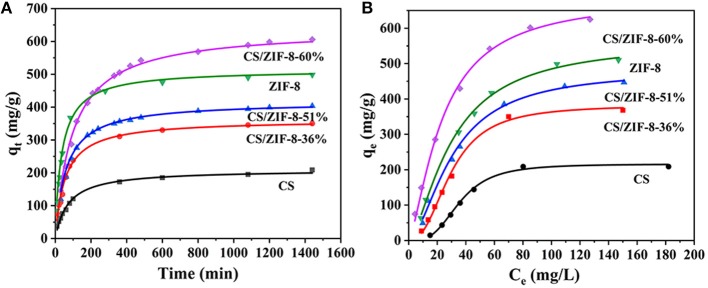
Kinetic and isothermal studies of U(VI) adsorption process, fitted with second-order kinetics **(A)** and Langmuir models **(B)** respectively (*C*_0_ = 100 mg•L^−1^, pH = 3, t = 24 h, *m*_ads_/*V*_sol_ = 0.2 g•L^−1^).

The adsorption kinetics of CS, ZIF-8 and CS/ZIF-8 composite were studied with different contact time. As shown in Figure 6A, several curves have similar trends where a fast adsorption of uranium is observed at the initial 60 min, and followed by a slower adsorption period until an equilibrium of uranium adsorption is reached. It could be explained from this: U(VI) ions first diffuse into the porous CS/ZIF-8 composite beads and they are adsorbed by interior active sites with a slow process until most surface active sites are occupied (Wang C. H. et al., 2018); To further investigate the mechanism of adsorption process, the U(VI) adsorption behavior are fitted using kinetic models as shown in Equation (2) and (3) (Yang et al., 2013):

Pseudo - first - order:

(2) $$\begin{array}{r} \ln\left(q_{e}-q_{t}\right)=\ln\;q_{e}-k_{1}t \end{array}$$

Pseudo - second - order:

(3) $$\begin{array}{r} \frac{t}{\;q_{t}}=\frac{1}{k_{2}{q_{e}}^{2}}+\frac{t}{q_{e}} \end{array}$$

where *q*_e_ and *q*_t_ (mg•g^−1^) refer to the U(VI) maximum adsorption capacity and the adsorption capacity at *t* (min), respectively, *t* is contact time (min), and *k* (g•mmol^−1^•min^−1^) is the kinetic constant. The fitting results (Figure 6A) show that the degrees of linearity of fitted curves of pseudo-second-order model are more suitable than those of pseudo-first-order model, and the values of correlation coefficient (*R*^2^) of U(VI) fitted by pseudo-second-order model are higher than those of pseudo-first-order model (Table S1), indicating that the adsorption process is mainly chemical adsorption. The calculated *q*_e_ is close to the experimental value. With the increase of ZIF-8 content, the adsorption amount gradually increases, the adsorption amount of CS/ZIF-8–60% reaches to 608 mg•g^−1^, which is superior evidently to the ZIF-8 powder (498 mg•g^−1^) and CS (208 mg•g^−1^). This better adsorption performance of the CS/ZIF-8–60% composite for U(VI) may be ascribed to its pore structures (Wang C. H. et al., 2018).

#### Adsorption Isotherms of the CS/ZIF-8 Composite Beads

In order to investigate the maximum adsorption capacity of the CS/ZIF-8 composites to uranium, the adsorption isotherm experiments with various initial concentrations of uranium (20–200 mg•L^−1^) were carried out at room temperature. As shown in Figure 6B, the Langmuir and Freundlich models are used to quantitatively analyze the adsorption isotherms. The equations are as follows (Aguila et al., 2017):

Langmuir models

(4) $$\begin{array}{r} {\frac{1}{q_{e}}=\frac{1}{q_{m}}+\frac{1}{q_{m}K_{L}C}}_{e} \end{array}$$

Freundlich models

(5) $$\begin{array}{r} \log q_{e}=\frac{\log\;C_{e}\;}{n}+\log K_{F} \end{array}$$

where *q*_m_ (mg•g^−1^) refers to the maximum adsorption capacity, *q*_e_ is the amount of adsorbed uranium at equilibrium (mg•g^−1^), *C*_*e*_ is the equilibrium concentration (mg•L^−1^), *K*_L_ (mL•g^−1^) is involved in the affinity of the adsorbate with the adsorbent, *K*_F_ refers to the Freundlich constant, and *n* is the Freundlich exponent. The results suggest that equilibrium isotherm experimental data is well-described by the Langmuir model with higher correlation coefficient (*R*^2^; Table S2), demonstrating that this adsorption process is a monolayer chemical adsorption. The theoretical maximum adsorption capacity of 625 mg•g^−1^ for CS/ZIF-8–60% is consistent with experimental value 629 mg•g^−1^. Compared with other reported MOF-based composite materials, the CS/ZIF-8–60% exhibits a very high adsorption capacity in uranium extraction (Table 1).

**Table 1:** Comparison of the maximum adsorption capacity of CS/ZIF-8–60% with other MOF-based adsorbents.

**Absorbents**	**pH**	***q*_**m**_(mg/g)**	**Refs**
GO-COOH/UiO-66	8.0	1002	Yang et al., 2017
CS/ZIF-8–60%	3.0	629	This work
GO/ZIF-67-Ag	7.0	602.41	Guo et al., 2019
PPy@ZIF-8	3.5	534	Li et al., 2018b
ZIF-8/PAN	3.0	530.3	Wang C. H. et al., 2018
Fe_3_O_4_@ZIF-8	3.0	523.5	Min et al., 2017

#### The Recyclability of the CS/ZIF-8 Composite Beads

**Figure 7:**
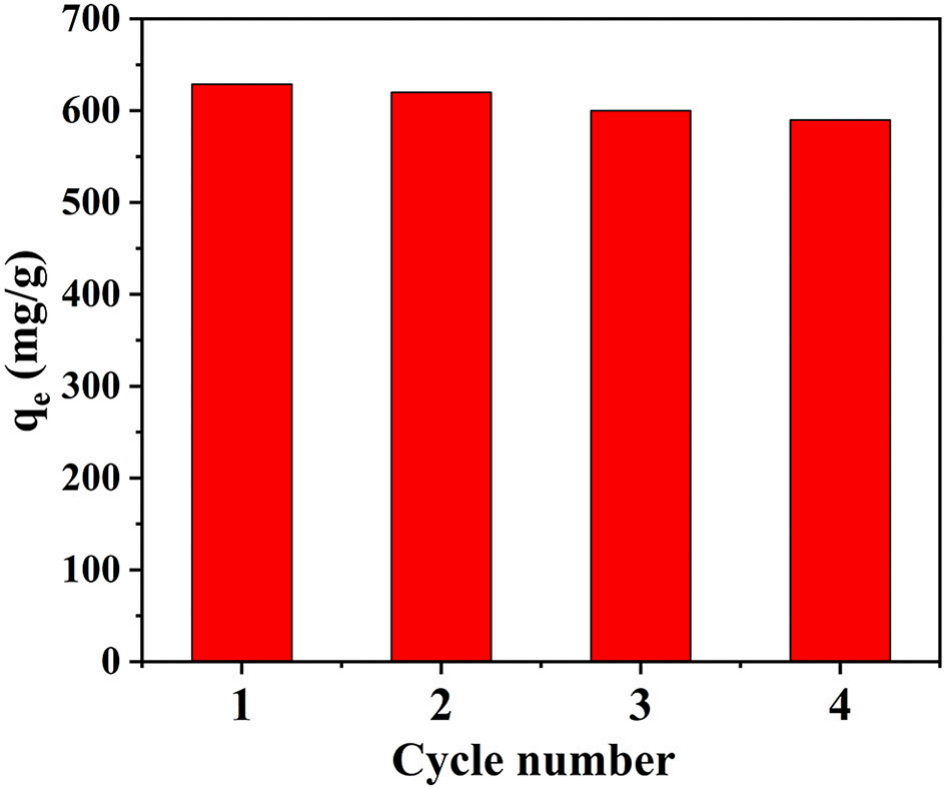
Elution cycle experiment of CS/ZIF-8–60% for U(VI) adsorption.

Reusability is a very important index for an adsorbent. A solution of NaHCO_3_ (0.1 M) was used as an eluent to evaluate the reusability of CS/ZIF-8 adsorbents. As shown in Figure 7, the CS/ZIF-8–60% can maintain a high adsorption performance after four adsorption/desorption cycles, specifying a good durability and recyclability, which is critical for the reduction of cost in practical uranium recovery applications. The slight decrease of the adsorption capacity could be caused by the inevitable mass loss of adsorbent during regeneration process. In addition, the structure of CS and ZIF-8 remained intact after the cycle experiment for uranium adsorption (Figure S3). Hence, the CS/ZIF-8 composite possess an excellent reusability and can serve as an economical and efficient adsorbent for the removal of U(VI) from aqueous solution.

### Potential Adsorption Mechanism

**Figure 8:**
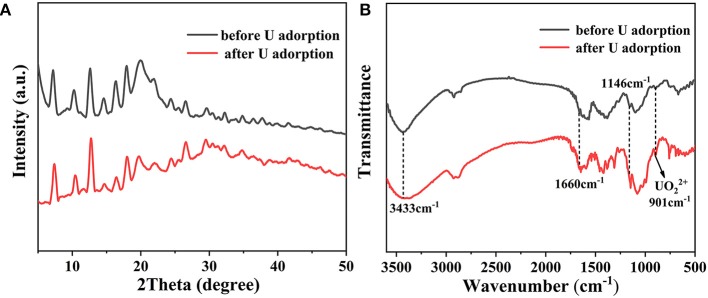
**(A)** PXRD patterns and **(B)** FT-IR spectra of CS/ZIF-8–60% before and after U(VI) uptake.

Additional characterization approaches were adopted to identify the underlying removal mechanism of U(VI). As shown in Figure 8A, the PXRD patterns before and after adsorption of uranium are consistent, indicating that no phase change occurs after adsorption. FT-IR studies show a characteristic absorption peak of uranyl appears at 901 cm^−1^ after uranium adsorption (Figure 8B). Moreover, both the vibrations of C-N at 1,146 cm^−1^ and NH_2_ at 1,660 cm^−1^ exhibit obvious red shifts after U(VI) uptake. Especially at 3,340 cm^−1^, the apparent broad peak is attributed to the stretching vibration of the hydroxyl group and the amino group, which suggests that there are a large amount of Zn-OH and N-H bonds through the water decomposition on the composite material surface. They are involved in the interaction with U(VI), proving the chelation of U(VI) ions with imidazole and chitosan (Cai et al., 2017).

**Figure 9:**
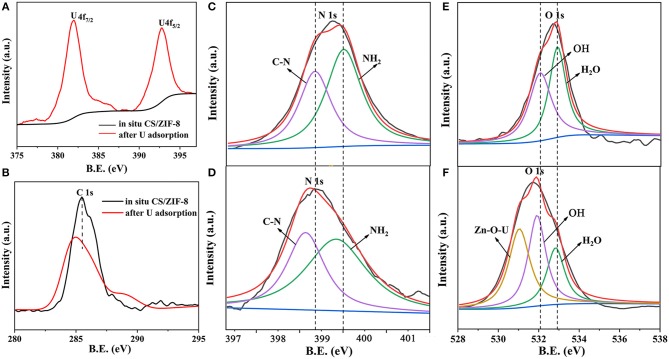
XPS survey scans of CS/ZIF-8–60% before and after U(VI) uptake; high-resolution XPS spectra of U 4f **(A)**, C 1s **(B)**, N 1s **(C,D)**, O 1s **(E,F)** before and after U(VI) uptake.

In order to better understand the adsorption mechanism of U(VI), XPS analysis was further carried out. The broad scan XPS spectrum of CS/ZIF-8–60% composite exhibits peaks of O 1s, C 1s, N 1s, and Zn 2p at 532.08, 281.08, 401.08, and 1022.08 eV, respectively (Figure S4). In addition, two distinct peaks of U 4f appear at 383.08 and 392.08 eV after U(VI) ingestion (Figure 9A). To verify the interaction between U(VI) and CS/ZIF-8 composite, narrow scans of C 1s, N 1s, and O 1s peaks are recorded and analyzed (Figures 9B–D). The spectral fitting shows that the energy peaks of C 1s and N 1s all exhibit a significant red shift after U(VI) adsorption, indicating the chelation of U(VI) with nitrogen from chitosan and imidazole (Wang C. H. et al., 2018). Figures 9E,F indicate an obviously difference of oxygen spectra. A new peak occurs with a binding energy of 530.75 eV representing Zn-O-U after uranium adsorption (Su et al., 2018). In addition, O-H has a weak red shift. These prove that hydroxyl groups on chitosan and Zn-OH moiety in ZIF-8 complex with uranyl (Su et al., 2018). The analysis of XPS is consistent with the above infrared experiment result.

## Conclusion

In summary, CS/ZIF-8 composite beads with different ZIF-8 loadings were synthesized by *in situ* growth for uranium removal. The maximum uranium adsorption capacity of CS/ZIF-8–60% is higher than most reported MOF-based composite adsorbents. In addition, the micron scale spherical adsorbent exhibits outstanding recyclability and is easy to recover. Based on the results of desorption experiments and spectroscopic analysis, the highly efficient removal mechanism of U(VI) is predominantly controlled by the -OH, -NH_2_, and C-N groups chelating with U(VI) ions. The results show that CS/ZIF-8 composite is a promising absorbent for uranium recovery from aqueous solution. The findings in this work will pave the way for the development of practical adsorbents for irradiative wastewater treatment.

## Data Availability

All datasets Synthesis procedure, uranium adsorption experiments and/or data processing, PXRD, SEM, TG, IR, ICP and XPS investigations for this study are included in the manuscript and/or the Supplementary Files.

## Author Contributions

WY and QP supervised the project. LL performed the experiments. DG participated the data analysis. XZ helped to analyze the results. LL wrote the manuscript with support from WY. All authors contributed to the general discussion.

### Conflict of Interest Statement

The authors declare that the research was conducted in the absence of any commercial or financial relationships that could be construed as a potential conflict of interest.
