# Supplementary material for: In situ Preparation of Chitosan/ZIF-8 Composite Beads for Highly Efficient Removal of U(VI)
Source: Front Chem. 2019 Sep 6;7:607. doi: 10.3389/fchem.2019.00607 (PMC6743043; doi:10.3389/fchem.2019.00607)
Supplement: Supplementary file 1 [file Data_Sheet_1.PDF]

Supplementary Material

Contents

Table S1. The adsorption kinetic fitting parameters**.**

Table S2. Langmuir and Freundlich Constants and Correlation Coefficients.

Figure. S1 CS/ZIF-8-60% after soaking in uranium aqueous solutions of different pH values for 3 days.

Figure. S2 DTA of CS/ZIF-8 composite beads with different content of ZIF-8.

Figure. S3 SEM of CS/ZIF-8-60% composite beads after uranium adsorption.

Figure. S4 The survey XPS spectra of the CS/ZIF-8-60% composite beads before and after adsorption of U (VI).

Table S1. The adsorption kinetic fitting parameters.

	Pseudo-second-order model			Pseudo-first-order model		
	R^2	q_e	K_2	R^2	q_e	K_1
CS	0.990	208.83	6.67×10^{-5}	0.962	190.903	1.11×10^{-2}
CS/ZIF-8-36%	0.989	350.192	4.95×10^{-5}	0.973	333.67	1.37×10^{-2}
CS/ZIF-8-51%	0.998	405.648	1.90×10^{-5}	0.963	382.865	6.34×10^{-3}
ZIF-8	0.995	498.448	2.8×10^{-5}	0.974	470.872	1.14×10^{-2}
CS/ZIF-8-60%	0.995	605.864	1.6×10^{-5}	0.975	573.74	7.21×10^{-3}

Table S2. Langmuir and Freundlich Constants and Correlation Coefficients.

	Langmuir adsorption			Freundlich adsorption		
	q_{max}	K_L	R^2	K_F	n	R^2
CS	208.83	0.000008	0.995	222.27	2.367	0.983
CS/ZIF-8-36%	368.096	0.0004	0.994	417.40	1.594	0.981
CS/ZIF-8-51%	446.8	0.0031	0.994	531.14	1.277	0.992
ZIF-8	510	0.0051	0.997	615.26	1.212	0.995
CS/ZIF-8-60%	625	0.0154	0.997	773.55	1.112	0.995

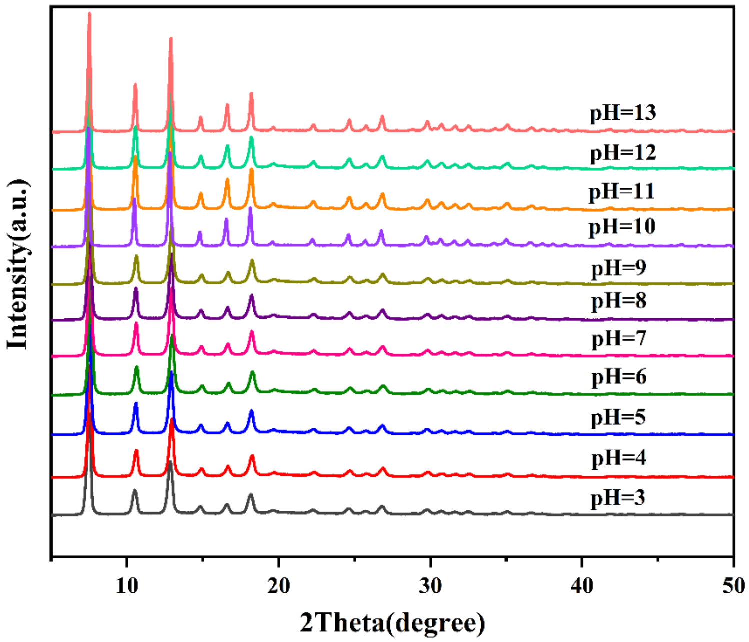

Figure S1. CS/ZIF-8-60% after soaking in uranium aqueous solutions of different pH values for 3 days

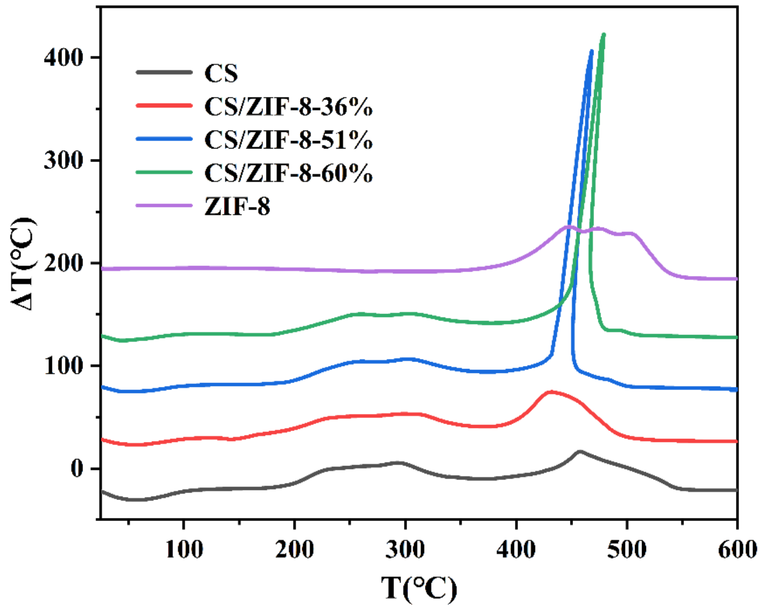

Figure S2. DTA of CS/ZIF-8 composite beads with different content of ZIF-8.

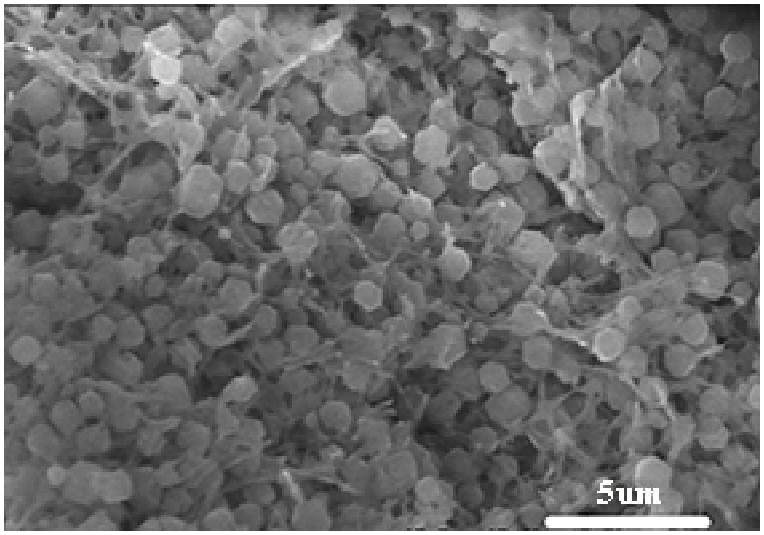

Figure S3. SEM of CS/ZIF-8-60% composite beads after uranium adsorption.

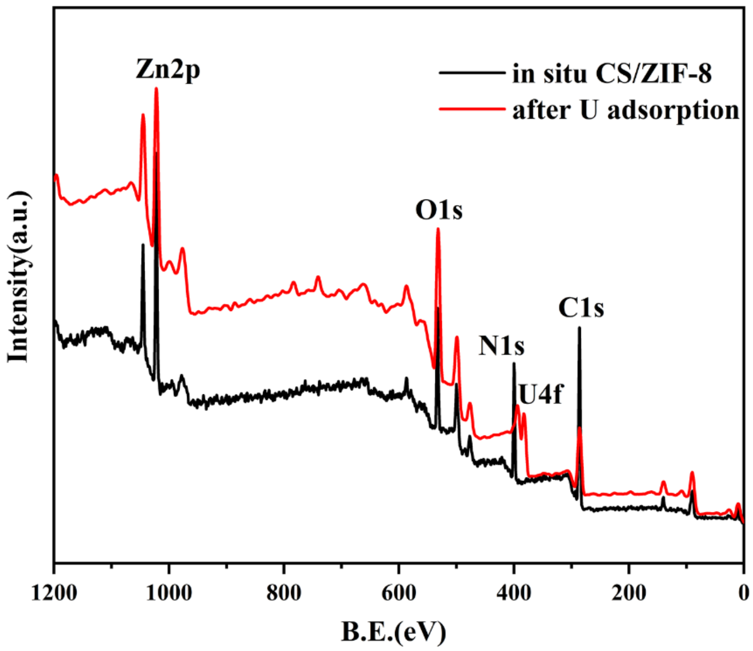

Figure S4. The survey XPS spectra of the CS/ZIF-8-60% composite beads before and after adsorption of U (VI).
